# Characterization of an Ex Vivo Equine Endometrial Tissue Culture Model Using Next-Generation RNA-Sequencing Technology

**DOI:** 10.3390/ani11071995

**Published:** 2021-07-03

**Authors:** Maithê R. Monteiro de Barros, Mina C. G. Davies-Morel, Luis A. J. Mur, Christopher J. Creevey, Roger H. Alison, Deborah M. Nash

**Affiliations:** 1 Institute of Biological, Environmental and Rural Sciences (IBERS), Aberystwyth University, Aberystwyth SY23 3FG, UK; mid@aber.ac.uk (M.C.G.D.-M.); lum@aber.ac.uk (L.A.J.M.); dmn@aber.ac.uk (D.M.N.); 2 Institute for Global Food Security, School of Biological Sciences, Queen’s University Belfast, Belfast BT7 1NN, UK; chris.creevey@qub.ac.uk; 3 Pathology Consultancy Services, Caerfyrddin Fach, Cilcennin, Lampeter SA48 8RN, UK; roger@rogeralison.com

**Keywords:** RNA-seq, endometritis, endometrium, equine, reproduction, explant, transcriptome, gene expression, tissue culture

## Abstract

**Simple Summary:**

Notwithstanding extensive research into fertility problems in mares, pregnancy rates have remained low mainly because of endometrial inflammation (endometritis). In the field of equine research, endometrial explants have been used to carry out in vitro studies of the mare’s endometrium. However, there has been no wide-ranging assessment of relative stability of this model over time. The aim of this study was to perform an in-depth transcriptomic assessment of endometrial explants over a culture period of 72 h and assess if they are representative of the whole mare. Explants at 24 h demonstrated significant changes when compared to biopsies at 0 h as expected. Even though gene expression changes were seen between 24 and 48 h of culture, prior to this window changes were dominated by the effects of explanting and culture and subsequently, transcription was generally compromised. Our results, therefore have defined the optimal period when explants can be used to study equine endometritis and how the endometrium is modulated during inflammation. It highlights the use of abattoir derived samples to understand the physiology and pathophysiology of the equine endometrium, negating the need to collect repeated uterine biopsies from living mares.

**Abstract:**

Persistent mating-induced endometritis is a major cause of poor fertility rates in the mare. Endometritis can be investigated using an ex vivo equine endometrial explant system which measures uterine inflammation using prostaglandin F_2α_ as a biomarker. However, this model has yet to undergo a wide-ranging assessment through transcriptomics. In this study, we assessed the transcriptomes of cultured endometrial explants and the optimal temporal window for their use. Endometrium harvested immediately post-mortem from native pony mares (*n* = 8) were sampled (0 h) and tissue explants were cultured for 24, 48 and 72 h. Tissues were stored in RNALater, total RNA was extracted and sequenced. Differentially expressed genes (DEGs) were defined using DESeq2 (R/Bioconductor). Principal component analysis indicated that the greatest changes in expression occurred in the first 24 h of culture when compared to autologous biopsies at 0 h. Fewer DEGs were seen between 24 and 48 h of culture suggesting the system was more stable than during the first 24 h. No genes were differentially expressed between 48 and 72 h but the low number of background gene expression suggested that explant viability was compromised after 48 h. *ESR1*, *MMP9*, *PTGS2*, *PMAIP1*, *TNF*, *GADD45B* and *SELE* genes were used as biomarkers of endometrial function, cell death and inflammation across tissue culture timepoints. STRING assessments of gene ontology suggested that DEGs between 24 and 48 h were linked to inflammation, immune system, cellular processes, environmental information processing and signal transduction, with an upregulation of most biomarker genes at 24 h. Taken together our observations indicated that 24–48 h is the optimal temporal window when the explant model can be used, as explants restore microcirculation, perform wound healing and tackle inflammation during this period. This key observation will facilitate the appropriate use of this as a model for further research into the equine endometrium and potentially the progression of mating-induced endometritis to persistent inflammation between 24 and 48 h.

## 1. Introduction

Persistent uterine inflammation (endometritis) has been extensively investigated in horses as it is the main cause of subfertility in mares and the third most important clinical problem affecting adult horses according to equine practitioners in the USA [1,2]. However, for many years pregnancy rates in broodmares have remained low, between 50% and 65% per oestrous cycle, despite extensive research into fertility problems [3,4]. Nearly 23,000 Thoroughbred (TB) mares were sent to the stud in Great Britain and Northern Ireland in 2019, yet only 61% produced a live foal [5]. It has been reported that 15–40% of a normal population of TB mares suffer from persistent endometritis, reducing pregnancy rates by 13% [6,7].

In the field of equine research, endometrial explants have been used to carry out many in vitro studies of the mare’s endometrium [8,9,10]. Tissue culture of explants has distinct advantages over isolated cell culture models. The technique saves time as cell culture requires several days or weeks to reach confluence [11]. Utilizing intact explants is particularly important. Most tissue explant models mechanically chop the tissue aiming for better oxygenation and perfusion of nutrients, but the chopping process itself leads to damage and disruption of tissue architecture [11]. By using intact explants, the extracellular matrix (ECM) remains intact which helps to maintain normal cell differentiation and functioning. Chopping will release damage-associated molecular patterns (DAMPS) which are intracellular and extracellular molecules typically released by the ECM after cell death and/or injury. DAMPS modulate the innate immune system, triggering inflammation even under sterile conditions [12,13], distorting the observations obtained because chopping heavily wounds the tissue. As a result, some ex vivo bovine studies have adopted an intact biopsy endometrium model to better mimic the whole cow [11,14]. Therefore, the present study used the equine endometrial explant culture proposed by Nash et al. [15], but substituted the mechanical tissue chopping by harvesting intact explants using sterile punch biopsies, as advocated by Borges et al. [11].

Harvesting endometrium from uteri collected at an abattoir negates the need to perform repeated uterine biopsies from living mares. Further, the large surface area of endometrium provided by each mare can be entirely harvested so that several different inflammatory stimuli and/or treatments of various concentrations can be concurrently studied, maximizing technical replication. An equine endometrial explant culture system composed of epithelial cells, stromal cells and resident leukocytes has been previously optimised. As with the whole animal, cultured explants respond to inflammatory stimuli by secreting markers of inflammation such as prostaglandin F_2α_ (PGF_2α_) [10,15,16]. Schwinghamer and colleagues [17] studied equine endometrial explants in culture for periods of 12, 24 and 48 h to investigate whether explants undergo significant changes during culture. They suggested that as early as 12 h of culture explants undergo cellular damage based on the increase of lactate dehydrogenase (LDH) activity, and at 48 h of culture light microscopy showed that some degenerative changes take place in the luminal epithelium and in the epithelium lining deeper endometrial glands. Nonetheless, there has been no assessment of global gene expression changes to intact equine endometrial explants during the culture period.

The aim was to undertake a transcriptomic assessment of ex vivo endometrial explants collected from native pony mares cultured over a period of 72 h. It assessed whether explants were representative of the whole mare in the pre-breeding, non-inflammatory state and established whether the transcriptome stabilizes once in culture. A temporal window relevant to persistent mating-induced endometritis was established in the current study, evidencing potential for using the endometrial explant tissue culture model for wider endometrium studies in horses. Therefore, the present study facilitates and informs the use of this model for a more in-depth study of equine endometritis to understand how the endometrium is modulated during inflammation.

## 2. Materials and Methods

### 2.1. Animals and Sample Collection

Uteri were collected from a random selection of native pony mares presented for euthanasia at a commercial abattoir for reasons unrelated to this study. Mares were slaughtered by free bullet followed by exsanguination of the jugular vein. Management of the animals, age or reproductive history were unknown. Corresponding blood samples were collected immediately after death from the jugular vein and stored in a plain vacutainer (367895, BD Vacutainer, Plymouth, UK). To estimate the stage of the oestrous cycle for each mare at the time of the death, uteri and cervices were physically and visually assessed as previously described by various authors [15,18,19]. Briefly, uteri and cervix were assessed for colour, appearance and tone whilst ovaries were immersed in a bucket with water and analysed using an ultrasound scanner for assessment of ovarian structures and follicle measurements (Table S1). The stage of oestrous cycle was retrospectively confirmed by measuring serum progesterone (P4) concentrations from the blood sample collected from each mare. Mares at the follicular phase of the oestrous cycle (*n* = 8) were utilized in this study to represent the stage of cycle that clinical endometritis is likely to occur. At the abattoir, endometrial biopsies were collected from each uteri within two hours of death, with the aid of a sterilized biopsy instrument (Equivet uterine biopsy forceps 62 cm, 141965, Kruuse, Guildford, UK), placed into RNALater (10437114, Fisher, Leicestershire, UK) and kept at 4 °C for 24 h during which samples were transported to the laboratory. After 24 h, RNALater was removed and tissues were stored at −80 °C until RNA extraction. These biopsy tissues were considered to be “0 h” endometrial samples in RNA-sequencing (RNA-seq) analysis. Uteri and blood samples were stored on ice and transported back to the laboratory within 6 h of collection. At the laboratory, serum P4 concentration was measured by an enzyme-linked immunosorbent assay (ELISA) kit (EIA 1561, DRG Diagnostics, Marburg, Germany) following the manufacturer’s instructions to confirm the phase of the oestrous cycle for each animal at the time of death.

### 2.2. Endometrial Cytology, Histology and Tissue Culture

To verify that uteri used were not inflamed, samples intended for cytological analysis were collected at the abattoir from each tract, using a sterile cytobrush (Cytobrush plus GT, C0112, Coopersurgical, Trumbull, CT, USA). Cytobrushes were rolled onto plain microscope slides and these were immediately airdried and subsequently fixed and stained with eosin and methylene blue (Shandon Kwik-Diff Kit, 9990700, ThermoFisher Scientific, Loughborough, UK). Specimens were evaluated by recording the number of neutrophils per high power field (hpf; 400×) across 10 fields [20,21]. Findings were classified as: normal (0 to 2 neutrophils/hpf), moderate inflammation (2 to 5 neutrophils/hpf) or severe inflammation (>5 neutrophils/hpf) [22,23,24]. Uteri showing moderate to severe inflammation were discarded from the study.

After transportation back to the laboratory, a biopsy was sampled from the uterine body and immediately placed in Bouin’s Fixative Fluid (10821910, Fisher, Leicestershire, UK). After fixation, tissues were subjected to several changes of 70% ethanol until their yellow colour disappeared. Tissues were then sectioned and placed into histological cassettes (U4635-1CS, Sigma Aldrich, Exeter, UK) and subjected to dehydration, clearing and infiltration. Sections of 8 μm were cut using a microtome (Minot 1212 rotary microtome, Ernst Leitz GmbH, Wetzlar, Germany) and transferred onto a microscope slide (10149870, Fisher, Leicestershire, UK) and placed on a hot plate at 40 °C to dry. Sections were de-waxed, followed by rehydration in descending concentrations of ethanol and stained with Harris haematoxylin and alcoholic 1% eosin for 5 min. Slides were mounted with Histomount solution (008030, Invitrogen, Paisley, UK) and a coverslip. Slides were left to dry and analysed under a light microscope. Histology slides were assessed by a Boarded Certified Veterinary Pathologist following the Kenney classification system [25] to indicate pathological or degenerative endometrial changes. As a result of the histological assessment, mares showing pathological or degenerative endometrial changes correspondent to category III were discarded from tissue culture. The Kenney classifications for the mares used in this study can be found in Table S2.

Working under aseptic conditions, punch biopsies were collected from the uterine horns (8 mm Kai Biopsy punch, BP-80F, Northumbrian Medical Supplies, Newcastle Upon Tyne, UK), immediately placed in warm supplemented Hank’s Buffered Saline Solution (HBSS) (14175053, Fisher, Leicestershire, UK) and left in the incubator (5% CO_2_, 38 °C) while biopsies from other uteri were harvested. Once biopsies were collected from all uteri, the tissue culture was assembled in a laminar flow hood (Microflow Biological, Bioquell, Andover, UK). Biopsy tissues were washed twice in un-supplemented HBSS and cultured by placing individual biopsies into one well of a six-well culture plate (10578911, Fisher, Leicestershire, UK). By following previously described protocols [11,15], biopsies were placed at the bottom of the well or on top of a lens-tissue lined wire platform and cultured in 3 mL and 4.25 mL of supplemented William’s Liquid E Medium (William’s phenol red free, 500 mL, A12176-01, Fisher, Leicestershire, UK), respectively. Explant cultures were performed in triplicate for each timepoint per animal. For all experiments, tissue explants were incubated at 38 °C in 5% CO_2_ in air. After periods of 24, 48 and 72 h explants were removed from the well and stored in 1.5 mL of RNALater for a 24 h period at 4 °C. After 24 h in RNALater explants were removed and stored at −80 °C until RNA extraction. William’s Liquid E Medium (500 mL) was supplemented with 0.01 µg/mL epidermal growth factor (EGF) (10605-HNAE-250, Fisher, Leicestershire, UK), 0.1 mg/mL neomycin (N6386, Sigma, Exeter, UK) and streptomycin (S6501, Sigma, Exeter, UK) solution, 2 mM of L-Glutamine (25030-032, ThermoFisher Scientific, Loughborough, UK), 2.5 µg/mL amphotericin B (A2942-20ML, Sigma, Exeter, UK), 5 mL of insulin-transferrin-selenium (ITS) (10524233, Fisher, Leicestershire, UK) and 50 mL of batch tested foetal bovine serum (FBS) (Invitrogen FBS, 10695023, Fisher, Leicestershire, UK). The supplemented medium was stored at 4 °C for a maximum period of 7 days. HBSS (100 mL), was supplemented with 0.1 µg/mL EGF, 1 mL of 10 mg/mL neomycin and streptomycin solution, 2 mM of L-Glutamine, 2.5 µg/mL Amphotericin B, 1 mL of ITS and 10 mL of FBS. Supplemented HBSS was stored at 4 °C for a maximum of 2 days.

### 2.3. RNA Extraction, Quantification and Sequencing

Total RNA was extracted from endometrial samples using an RNA purification kit (GeneJET RNA Purification Kit, Thermo Scientific, Bishops Stortford, UK) following the manufacturer’s instructions. The NanoDrop 1000 Spectrophotometer (Thermo Scientific, UK) was used to check RNA concentration and purity. In addition, RNA samples were fractionated on a 1% agarose gel to assess RNA integrity which was indicated by visualization of distinct 28S and 18S ribosomal RNA bands.

Complementary DNA (cDNA) synthesis, fragmentation and library preparation were performed using the Illumina TruSeq Stranded mRNA kit (20020594, Illumina, San Diego, CA, USA) according to the manufacturer’s instructions to prepare the dual-indexed next-generation sequencing libraries. Briefly, from each sample poly-A messenger RNA (mRNA) was purified, fragmented, and reversed transcribed into cDNA. The cDNA was end-repaired and connected to adaptors that contained unique indexes for each sample. The connected products were amplified by a polymerase chain reaction (PCR) (98 °C for 30 s, 15 cycles of 98 °C for 10 s, 60 °C for 30 s, 72 °C for 30 s, 72 °C for 5 min and then cooling to 4 °C), followed by purification with AMPure (A63882, Beckman Coulter, High Wycombe, UK) to remove PCR reagents and adaptor dimers. Product quantity in ng/µL was assessed using a Qubit fluorescence spectrophotometer (Thermo Fisher Ltd.). The final library dilution was 8 pM. The diluted library was loaded, bound, and amplified onto a flowcell using an Illumina cBOT platform and then transferred to the HiSeq2500 and paired-end sequencing runs in the 2 × 216 bp format.

### 2.4. Data Processing and Gene Expression Analysis

The pair-end sequencing produced forward and reverse reads for each sample, totalling 64 raw reads. Raw reads were subjected to quality analysis using FastQC software (version 0.11.2) [26] and filtering using Trimmomatic software (version 0.33) [27] for sequencing adapters removal (i.e., ILLUMINACLIP), bases were removed from the beginning and end of the reads if the quality was below 30 (i.e., LEADING or TRAILING = 30). A sliding window trimming approach was performed once the average quality within the window fell below 30 (i.e., SLIDINGWINDOW 4:30), reads were dropped if they were below a specific length of 100 bp (i.e., MINLEN = 100), and a specified number of 10 bases from the start of the read were removed (i.e., HEADCROP = 10). The quality of the trimmed reads was assessed again by FastQC. Reads were mapped to the Equus caballus reference genome (Ensembl, EquCab3.0; GCA_002863925.1) using Bowtie software (version 2.2.3) [28] and TopHat software (version 2.0.14) [29]. The number of reads/fragments assigned to genomic features for each sample and the Equus caballus annotation file available from Ensembl website (version EquCab3.0) using the FeatureCounts software (version 1.5.2) [30]. Sequencing data are provided in Table S3. The concordant pair alignment rate to the annotated equine genome from all samples was between 86.2% and 93.6%.

Differentially expressed genes (DEGs) were identified using the DESeq2 R/Bioconductor version 1.28.1 [31,32]. Benjamini-Hochberg (BH) false discovery rate (FDR) method [33] was performed in R (version 4.0.3) to correct for multiple testing. Genes were considered differentially expressed at an FDR of 0.05 with a Log_2_ fold change (Log_2_FC) greater or equal to ± 2. DEGs were submitted to the Search Tool for the Retrieval of Interacting Genes/Proteins (STRING) database [34] (http://string-db.org, accessed on 5 February 2021) for network analysis and gene associations. Kyoto Encyclopedia of Genes and Genomes (KEGG) pathways [35] (http://www.genome.jp/kegg, accessed on 9 February 2021) were also used to identify significantly enriched pathways. Principal component analyses (PCA) and heatmaps were generated using R (version 4.0.3).

## 3. Results

**Figure 1:**
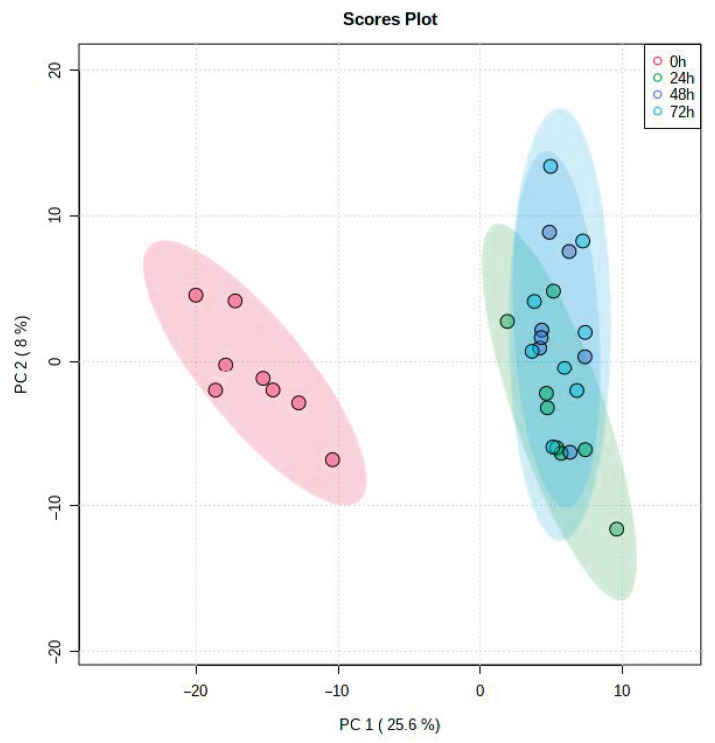
PCA plot of the gene expression profiles for the eight horses. Biopsies taken at 0 h and explants cultured up to 24, 48 and 72 h (*n* = 8). The analysis demonstrates clustering for the 0 h (control, representing the whole mare) and another cluster for the cultured samples (24, 48 and 72 h time points). The paler ellipses represent 95 % confidence intervals for each experimental class.

The global gene expression of equine endometrial explants (*n* = 8 horses) cultured in vitro for periods of 24, 48 and 72 h were compared to autologous ex vivo 0 h biopsies, representing the whole mare. Patters of gene expression were indicated by PCA based on all gene expression, not solely DEGs (Figure 1). Samples obtained at 0 h were separated from all others across the major source of variation along PC1. There was lesser variation along PC2 with the samples with the 24 h samples being discrete from the 48 and 72 h group which could not be distinguished.

**Figure 2:**
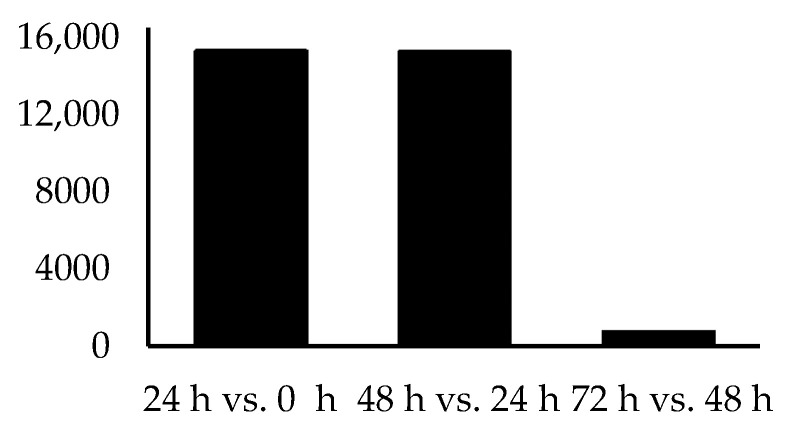
Comparison of all genes expressed at all time points. Pairwise comparison of the number of genes expressed at the different time points (0, 24, 48 and 72 h) after mapping RNA-sequencing reads to the equine reference genome.

**Figure 3:**
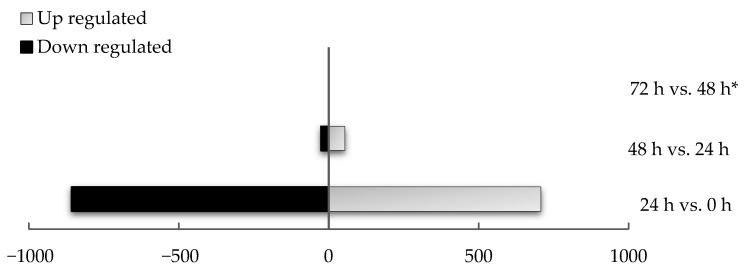
Comparison of differentially expressed genes at all time points. Comparison of up and downregulated genes between all-time points (*p* < 0.05; Log_2_FC ≥ ± 2). * The comparison between 48 and 72 h of culture did not retrieve any DEG.

To identify the sources of variation, DEG analyses were undertaken, comparing between time points. When comparing the transcriptomes of explants cultured for 24 h to those of whole mares’ controls (0 h), 707 genes were significantly differentially upregulated and 859 downregulated. In contrast, when comparing explant tissues cultured for 48 h relative to 24 h, only 53 genes were significantly upregulated while 27 genes were downregulated. Finally, the transcriptome of explants cultured for 72 h revealed no differentially expressed genes compared to 48 h samples. Table 1 compares the number of expressed genes (background genes) and the number of DEGs between timepoints, whilst Figure 2 and Figure 3 graphically represent data featured in Table 1. It should be noted that unlike the 0–48 h period relatively few genes were expressed within the 48–72 h window.

Following the STRING functional enrichment analysis of DEGs between 0 and 24 h, a total of 41 KEGG pathways were found to be significantly enriched (Table S4). Table 2 features KEGG pathways that are relevant to the current study. These indicated prominent changes linked to inflammation, immune system, cellular processes, environmental information processing and signal transduction. STRING enrichment analysis of DEG between 24 and 48 h retrieved a total of five significantly enriched KEGG pathways (Table 3). These suggested cell cycle events, cellular processes, and ECM remodelling.

**Figure 4:**
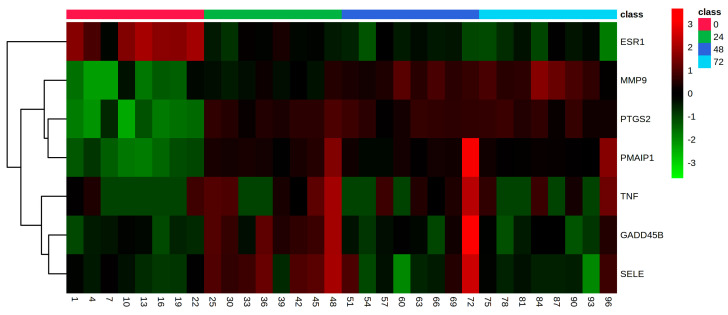
Heatmap featuring the gene expression pattern of genes of interest between 0, 24, 48 and 72 h of culture.

Genes previously used as biomarkers of endometrial function [17] were also studied to better understand explants’ functionality once in culture. The oestrogen receptor, *ESR1*, was upregulated at 24 h vs. 0 h (FDR = 1.48 × 10^−15^; Log_2_FC = −2.5), however it was not differentially expressed at 48 vs. 24 h (FDR = 0.35; Log_2_FC = 0.5). Whilst *PTGS2*, prostaglandin-endoperoxide synthase 2, was upregulated at 24 h vs. 0 h (FDR = 1.97 × 10^−27^; Log_2_FC = 6), it was not differentially expressed at 48 h vs. 24 h of culture (FDR = 0.99; Log_2_FC = −0.01). The possible initiation of cell death was considered by examining the expression of *GADD45B* (growth arrest and DNA-damage-inducible beta I) and *PMAIP1* (phorbol-12-myristate-13-acetate-induced protein 1) [17]. *GADD45B* was not differentially expressed at 24 h (FDR = 3.2 × 10^−4^; Log_2_FC = 1.6) nor at 48 h (FDR = 0.35; Log_2_FC = −0.6) using the usual comparisons. *PMAIP1* was upregulated at 24 h (FDR = 1.64 × 10^−13^; Log_2_FC = 3.1) but not differentially regulated at 48 h of culture (FDR = 0.79; Log_2_FC = 0.2). Figure 4 displays a heatmap showing the expression pattern (count matrix) of the biomarker genes: *ESR1, MMP9, PTGS2, PMAIP1, TNF, GADD45B* and *SELE.* This indicted a transient increase in expression by most biomarkers at 24 h whilst *ESR1* was suppressed. At 48 and 72 h consistent elevated expression of *MMP9,* a matrix metallopeptidase, was seen in each horse explant. Expression of *PTGS2* (prostaglandin processing gene) and *PMAIP1* was induced at 24 h and maintained in each horse explant up to 72 h. Correlation analyses indicated that *ESR1* was inversely correlated with all biomarker genes and most clearly with *PTGS2* (Figure S1). In turn, *PTGS2* was positively correlated with all biomarker genes but *ESR1.*

## 4. Discussion

Endometritis is a major cause of infertility in mares. Whilst endometritis has been the subject of many studies that investigate pathophysiology, the development of a viable explant model that maintains multicellular integrity would represent an important advance to facilitate further study of the equine endometrium and better understand endometritis. Preliminary approaches to develop such a model system have been proposed but these lacked the extensive validation that is possible using transcriptomic approaches. In this study, we describe distinctive phases of transcriptomic responses during explant culture.

In the present study, the upregulation of *ESR1* at 24 h of culture implied that explants cultured for the first 24 h cannot be used to study oestrogen-responsive genes [17]. However, *ESR1* is not differentially regulated at 48 h of culture, implying that explants can indeed be used in the study of oestrogen responsiveness in the window of 24–48 h of culture. Along with *ESR1, PTGS2* was also used as a biomarker of endometrial function as it encodes an enzyme that plays a role in the conversion of arachidonic acid to prostaglandin endoperoxide, which is an endometrial biomarker of inflammation [17,36]. *PTGS2* upregulation at 24 h of culture indicates arachidonic acid processing, possibly linked to inflammatory events during the first hours of culture due to explant removal from uterus. Nonetheless, *PTGS2* was not differentially regulated at 48 h of culture, possibly indicating that at this time inflammation was stable. These findings might suggest that this tissue model offers a short window of 24–48 h when further experiments which use challenged explants to investigate endometritis-like events can be performed as suggested by Schwinghamer et al. [17].

Growth arrest and DNA-damage-inducible beta I gene, *GADD45B*, that regulates growth and apoptosis [37] was not regulated at 24 h nor at 48 h of culture. *PMAIP1* (Phorbol-12-Myristate-13-Acetate-Induced Protein 1) which encodes a pro-apoptotic protein [38] was upregulated at 24 h of culture but it was not differentially regulated at 48 h. As previously described by Schwinghamer et al. [17], both *GADD45B* and *PMAIP1* can be used as biomarkers of cellular death in equine endometrial explant systems. This implied that cell death was triggered during the first 24 h of culture, but that apoptosis was not likely to happen at 48 h. These observations also support relative stability in the explants in the 24–48 h time frame. However, by 72 h only a very low number of genes were expressed (804 at 72 h vs. 15290 at 48 h; Table 1) which aligns with Schwinghamer et al. [17] observations that degenerative changes occur in cultured explants after 48 h of culture. Thus, our transcriptomic experiments suggest a relatively short window of 24–48 h to analyse endometrial tissue and to study endometritis using the explant system in the future.

During the initial 0–24 h culture phase there were a series of transcriptional changes that were consistent with stress. The TNF signalling pathway, that plays a role in inflammatory cell proliferation, differentiation, survival and cell death [39], was upregulated at 24 h of culture (Figure S2). *TNF* induces the expression of E-selectin, *SELE*, that is involved in the localization and development of inflammation [40,41]. The upregulation of *SELE* is consistent with the upregulation of TNF-mediated inflammation during the first 24 h of culture seen in the TNF signalling pathway. Increases in pro-inflammatory interleukin-1β (*IL–1β*) and chemokine C-C motif ligand 20 (*CCL20*) also suggests that inflammation was a feature of the explant system. Therefore, such observations are consistent with inflammatory events being prominent in the explant culture during the first 24 h. The upregulation of *MMP9* should also be seen as wider change in the ECM that features in our transcriptome during the first 24 h of culture. It is involved in the breakdown of ECM and other normal physiological and pathological processes [42,43,44]. *MMP9* has been linked to a reduction in connective tissue deposition, in restoring microcirculation and it also plays a role in wound repair [42,45,46]. The upregulation of *MMP9* seen at 24 h suggests that the explants have started healing wounds caused by mechanical removal from the uterus and that they are trying to restore microcirculation to support their survival in the tissue culture environment.

The ECM is a network of collagen, fibronectin, enzymes, proteoglycans and glycoproteins [47,48,49] that is continually remodelled to control tissue homeostasis, cell growth, migration, differentiation and morphogenesis [50,51]. ECM molecules are then able to control the magnitude of inflammation based on their ability to bind to different pattern recognition receptors (PRRs). Equally, immune cells release enzymes and inflammatory mediators which can further degrade ECM or can alter its composition [52,53,54]. In our explant system, most collagen coding genes were downregulated (*COL17A1, COL21A1, COL2A1, COL9A1, COL26A1, COL1A2, COL4A4* and *COL4A6*) and only two were upregulated (*COL12A1* and *COL20A1*) at 24 h. Type IX collagen plays an important role in the formation of a collagen network and it maintains cartilage integrity and organization [55], which might suggest that at 24 h of culture explants are building a collagen network to recover from mechanical trauma. Downregulation of type IV collagen leads to an upregulation of *MMP9* and thus related to cell migration and invasion [56]. *COL12A1* has been associated with ligament ruptures in women [57], therefore its upregulation at 24 h of culture might be related with tissue rupture after excision from the uterus. On the other hand, type XX collagen is still not well understood and its function is relatively unknown [58].

An interplay between pro- and anti-inflammatory events is also suggested by examination of interleukin (IL) expression patterns in the cytokine–cytokine receptor interaction pathway (Figure S3). Most ILs were found to be upregulated at 24 h of culture (*IL1A, IL1B, IL1R2, IL1RN, IL21R, IL23A, IL24, IL2RA, IL31RA, IL36A, IL36G, IL4R* and *IL6)* whilst *IL25* (also known as *IL17E*) was the only IL shown to be downregulated. It has been shown that during gut inflammation the production of IL25 protein is diminished [59]. This can suggest that during the first 24 h of culture the explants are indeed undergoing inflammation, thus the downregulation of *IL25.* The *IL1* family group induces a network of proinflammatory cytokines, which regulate and initiate inflammatory responses [60]. *IL24* belongs to the *IL10* family, and is released by immune cells such as monocytes, macrophages and T cells [61]. *IL24* is suggested to control cell proliferation and survival and it participates in wound healing [62]. *IL23A* is a protein encoded by *IL23* that plays an important role in inflammatory responses. It also increases angiogenesis and upregulates *MMP9* [63] as seen in this explant tissue model. Most ILs found to be upregulated at 24 h of culture were from the IL1 family, demonstrating that inflammation was a feature of explants during the first hours of culture. *CCR7,* C-C motif chemokine receptor 7, was upregulated at 24 h of culture and the C-C chemokine ligand family (CCLs) were differentially expressed. *CCL2, CCL11, CCL20, CCL26* and *CCL27* were all upregulated whilst *CCL24* was downregulated. During physiological and pathological conditions, specially during inflammation, CCLs play an important role in the recruitment of immune cells [64]. *CCL24* is also involved in pathological processes such as inflammation and fibrosis, but it has been found that *CCL24* blockade can be used therapeutically to inhibit cell activation and pulmonary inflammation [65]. Therefore at 24 h there could be a recruitment of immune cells due to the local inflammation produced after explant removal from uteri by CCLs. These events should be considered as part of a modulatory event influencing explant cell growth in culture.

When comparing the transcriptome at 48 to the 24 h, *IL23A* and *IL24* were downregulated whilst *CSF3R* was upregulated in the cytokine–cytokine receptor interaction pathway. *CSF3R*, colony-stimulating factor 3 receptor, is involved in the production, differentiation and function of granulocytes and it also plays a role in cellular processes such as surface adhesion and recognition [66,67]. A downregulation of interleukin-coding genes leads to the hypothesis that the inflammatory process due to tissue injury was mounted, tackled and it was returning to normal by 48 h of culture. At this stage the only TNF signalling pathway output that was modulated was *MMP9*. As outlined, *MMP9* is a pro-angiogenic factor that is involved in ECM remodelling and that coordinates epithelial wound repair by removing fibrinogen matrix and stimulating collagen contraction [42,46]. Therefore, wound repair is still likely to be a feature of explants at 48 h. At 48 h of culture collagen (*COL1A2* and *COL24A1)* and fibronectin *(FN1)* genes were also upregulated when compared to the transcriptome of explants cultured for 24 h. Fibronectin binds cells with collagen fibres and cell-surface integrins and is involved in cell adhesion, growth, migration, differentiation, and other processes such as wound healing [68]. As fibronectin expression is directly linked to epithelial cell growth [69], it would implicate explant cell growth at 48 h of culture.

A limitation of this work was undoubtedly the lack of medical history for the animals from which uteri were collected as these were sourced from a commercial abattoir. It was important that uteri used did not have any inflammation at the time of collection that may adversely affect the data. As such the uteri collected were from native pony mares to represent a population that had not been intensively managed. These ponies were collected for commercial purposes unrelated to our study, from semi-feral populations that were rounded up from the mountains of South Wales, UK, in the days before being transported to the abattoir. Unlike domestic horses being sent to the abattoir, these were not being slaughtered due to old age, injury or illness, but were otherwise healthy individuals, less likely to have underling inflammatory processes present at the time of death. However, as these semi-feral populations receive minimal management or intervention, no medical history was documented or available to this project. In terms of uterine health specifically, we insured that uterine inflammation was not apparent at the time of tissue collection using cytological and histological assessment of each uterus used. Any animal that had evidence of inflammation using these techniques was retrospectively disregarded from the study. The cytological and histological analyses conferred confidence that, without a detailed medical history, the uteri of these mares were not undergoing inflammation at the time of tissue collection.

## 5. Conclusions

This study provides the first comprehensive in vitro assessment of the pre-breeding, non-inflammatory-challenged, global gene expression analysis of cultured equine endometrial explants and autologous ex vivo biopsies. It highlights the utility of abattoir-derived samples to understand the physiology of the equine uterus. The upregulation of interleukins, *ESR1, PTGS2, PMAIP1, SELE* and *TNF* is indicative that explants were undergoing stress and inflammatory events during the first 24 h of culture. Nonetheless, at 24 h explants also showed an upregulation of *MMP9*, which suggests that explants were undergoing microcirculation restoration and wound healing after removal from the uterus. The downregulation of interleukin-coding genes and an upregulation of *MMP9* seen at 48 h of culture suggests that inflammatory processes were mounted and tackled and that the tissue was restoring homeostasis between 24 to 48 h of culture. *GADD45B* and *PMAIP1* were not differentially expressed at 48 h of culture, indicating that cell death was not occurring at a significant level at this time. At 72 h of culture only a limited number of genes were expressed by the explants when compared to the other timepoints, suggesting that degenerative changes take place in the tissue after 48 h of culture.

We suggest that cultured explants may be a suitable exemplar to further study the equine endometrium with particular application for modelling equine mating-induced endometritis progression to persistent inflammation between 24 and 48 h once in culture. Following future validation at the protein level, the explant tissue culture model may be used for wider studies applied to endometritis in the mare and potentially also in dogs and pigs as well as for investigating conditions underlying the endometrium.

## Figures and Tables

**Table 1:** Comparison of gene counts and differentially expressed genes (DEGs) (FDR < 0.05 and Log_2_FC ≥ ±2) and the number of up and downregulated genes across all time point comparisons.

Time Point Comparison	Number of Genes Expressed	Number of DEGs	Upregulated Genes	Downregulated Genes
24 h vs. 0 h	15,326	1566	707	859
48 h vs. 24 h	15,290	80	53	27
72 h vs. 48 h	804	0	0	0

**Table 2:** The seven most important KEGG pathways when comparing the transcriptome at 24 h relative to the transcriptome at 0 h. Table created using the STRING website.

Pathway Code ^a^	Description ^b^	Count ^c^	FDR *p*-Value ^d^
4610	Complement and coagulation cascades	27	1.27 × 10^−9^
4512	ECM-receptor interaction	27	3.74 × 10^−7^
4066	HIF-1 signaling pathway	29	5.20 × 10^−7^
4060	Cytokine-cytokine receptor interaction	43	7.61 × 10^−7^
4151	PI3K-Akt signaling pathway	55	2.48 × 10^−5^
4510	Focal adhesion	39	1.58 × 10^−4^
4668	TNF signaling pathway	22	2.12 ×10^−3^

^a^ KEGG pathway identification code. ^b^ Description of each KEGG pathway. ^c^ The number of genes involved in each pathway. ^d^ Overrepresented *p*-value with false discovery rate (FDR) statistical correction for multiple comparisons.

**Table 3:** Summary of the five enriched KEGG pathways when comparing the transcriptome at 48 h relative to the transcriptome at 24 h. Table created using the STRING website.

Pathway Code ^a^	Description ^b^	Count ^c^	FDR *p*-Value ^d^
4110	Cell cycle	6	0.007
4114	Oocyte meiosis	5	0.016
4630	Jak-STAT signalling pathway	5	0.036
4512	ECM-receptor interaction	4	0.040
4914	Progesterone-mediated oocyte maturation	4	0.040

^a^ KEGG pathway identification code. ^b^ Description of each KEGG pathway. ^c^ The number of genes involved in each pathway. ^d^ Overrepresented *p*-value with false discovery rate (FDR) statistical correction for multiple comparisons.

## Data Availability

The data presented in this study are openly available in the Gene Expression Omnibus (GEO) repository at the National Center for Biotechnology Information (NCBI), accession number GSE169759.

## Supplementary Materials

The following are available online at https://www.mdpi.com/article/10.3390/ani11071995/s1. Table S1: Characterization of the phase of the oestrous cycle based on an ultrasonic view of ovaries and physical aspect of the cervix and progesterone analyses, Table S2: Kenney classification of mare’s endometrium, Table S3: Summary of the 32 RNA-sequencing samples, Table S4: Summary of the 41 enriched KEGG pathways when comparing the transcriptome at 24 h relative to the transcriptome at 0 h, Figure S1: Pearson correlations between endometritis associated biomarkers genes focusing on (a) *ESR1* and (b) *PTGS2**,* Figure S2: DEGs in the tumor necrosis factor (TNF) signalling pathway between 24 and 0 h (control), Figure S3: DEGs in the cytokine–cytokine receptor interaction pathway between 24 and 0 h (control).
