# Supplementary material for: Characterization of an Ex Vivo Equine Endometrial Tissue Culture Model Using Next-Generation RNA-Sequencing Technology
Source: Animals (Basel). 2021 Jul 3;11(7):1995. doi: 10.3390/ani11071995 (PMC8300099; doi:10.3390/ani11071995)
Supplement: Supplementary file 1 [file animals-11-01995-s001.zip › Supplementary File 1 - Table S1 - Characterization of the phase of the oestrous cycle.pdf]

Table S1

Characterization of the phase of the oestrous cycle based on an ultrasonic view of ovaries and physical aspect of the cervix and progesterone analyses

Stage of the cycle	Ovaries	Cervix	Progesterone concentration
Follicular	One or occasionally two dominant follicles (>35 mm in diameter), absence of corpus luteum, follicle wall increasingly echogenic and follicle changing from spherical to irregular shape before ovulation.	Cervix is red in colour, soft, open and with a ‘rose flower’ characteristic. Two to three fingers can be passed through the cervix.	< 2 ng/mL
Luteal	Follicles between 5 to 25 mm in diameter, increasing in size as luteal phase progresses. Presence of corpus luteum.	Cervix is pale, closed and with a ‘close bud’ characteristic. No finger can be passed through cervix at early luteal phase, whereas one to two fingers can be passed through at late luteal phase.	> 2 ng/mL
Anoestrous	Few small follicles (<15 mm). Absence of corpus luteum.	Cervix is pale in colour. Little cervical tone, one finger can be passed through the cervical os.	< 1 ng/mL
