# Supplementary material for: Characterization of an Ex Vivo Equine Endometrial Tissue Culture Model Using Next-Generation RNA-Sequencing Technology
Source: Animals (Basel). 2021 Jul 3;11(7):1995. doi: 10.3390/ani11071995 (PMC8300099; doi:10.3390/ani11071995)
Supplement: Supplementary file 1 [file animals-11-01995-s001.zip › Supplementary File 2 - Table S2 - Kenney Classification of Mare's endometrium.pdf]

Table S2

Kenney Classification of Mares' Endometrium

Mares	Histology report	Kenney classification
1	Medium epithelium. Stratum compactum examined, no pathologic findings noted. Stratum spongiosum showed difuse grade 1 fibrosis.	I
2	Medium epithelium. Stratum compactum showed lymphocyte focal infiltration grade 1. Stratum spongiosum examined, no pathologic findings noted.	I
3	Medium epithelium. Stratum compactum examined, no pathologic findings noted. Stratum spongiosum showed focal cystic glands grade 1.	I
4	Tall epithelium. Stratum compactum showed diffuse gland atrophy grade 1. Stratum spongiosum showed diffuse gland atrophy grade 2, multifocal cystic glands grade 3 with inspissated contents, fibrosis periglandular grade 3 and lymphocyte infiltration grade 1.	II
5	Tall epithelium. Stratum compactum showed lymphocyte infiltration grade 1. Stratum spongiosum showed focal cystic glands grade 1.	I
6	Medium epithelium. Stratum compactum showed lymphocyte infiltration grade 1, macrophage and erythrophagocytes infiltration grade 2. Stratum spongiosum showed lymphocyte infiltration grade 1, macrophage and erythrophagocytes infiltration grade 2 and siderocyte infiltration grade 1.	I
7	Tall epithelium. Stratum compactum showed focal lymphocyte infiltration grade 1. Stratum spongiosum examined, no pathologic findings noted.	I
8	Stratum compactum showed infiltration of macrophages and erythrophagocytes grade 3. Stratum spoingiosum showed focal cystic glands grade 1, focal periglandular fibrosis grade 1 and infiltration of macrophages and erythrophagocytes grade 1.	I

[a] Grade 1 = minimal/very few/very small. Grade 2 = slight/few/small. Grade 3 = moderate/moderate number/moderate size. Grade 4 = marked/many/large.
