# Supplementary material for: Characterization of an Ex Vivo Equine Endometrial Tissue Culture Model Using Next-Generation RNA-Sequencing Technology
Source: Animals (Basel). 2021 Jul 3;11(7):1995. doi: 10.3390/ani11071995 (PMC8300099; doi:10.3390/ani11071995)
Supplement: Supplementary file 1 [file animals-11-01995-s001.zip › Supplementary File 3 - Table S3 - Summary of the 32 RNA-Sequencing samples.pdf]

Table S3
Summary of the 32 RNA-Sequencing samples

Horse	Total sequences Raw reads[a]	Sequences after trimming[b]	Per sequence GC content (%)	Aligned pairs[c]	Alignment rate[d]	Total count[e]
H1-0h	19717258	8502094	49	7358186	86.2%	5197583
H1-24h	67308470	32342562	50	2884440	88.9%	2180946
H1-48h	19839515	9954298	50	8838404	88.5%	6823863
H1-72h	14432128	7358130	50	6582181	89.1%	4900818
H2-0h	18178744	9085634	51	7880970	86.5%	5655934
H2-24h	12681202	6069612	50.5	5437735	89.3%	4184830
H2-48h	30143876	15499068	50	1411214	90.7%	1082339
H2-72h	12826353	6530807	49.5	5933521	90.4%	4429069
H3-0h	20960250	10117417	50	9154720	90.2%	6380111
H3-24h	15509308	7851773	50	7119652	90.4%	5369106
H3-48h	16948027	8578848	51	7836263	91.1%	5985166
H3-72h	25647761	13313650	50.5	1225096	91.6%	9310609
H4-0h	28670134	13715002	51	1194665	86.8%	8575720
H4-24h	20422712	10795659	51	9902068	91.4%	7521370
H4-48h	15788862	8014918	51	7388535	91.9%	5582761
H4-72h	18383955	9131700	51	8317166	90.8%	6402180
H5-0h	22947695	9857331	50.5	8960958	90.6%	6415387
H5-24h	21849937	10419618	51	9440102	90.3%	7128889
H5-48h	24772671	12453536	51	1146623	91.7%	8804639
H5-72h	23018161	11156075	51	1032539	92.2%	7980186
H6-0h	18112353	9450237	50	8529686	90.0%	6110302
H6-24h	18419612	10260890	51	9328179	90.6%	7078320
H6-48h	19130571	10543071	50	9721228	91.8%	7310548
H6-72h	18810862	10857330	50	1017581	93.3%	7657996
H7-0h	18438679	8526023	50	7893428	92.1%	5666816
H7-24h	20176785	10709870	50	1004868	93.5%	7528436
H7-48h	25947827	14121856	50.5	1313489	92.6%	1025650
H7-72h	21821418	11656927	51	1096165	93.6%	8616341
H8-0h	24180545	13500189	50	1252684	92.4%	9231933
H8-24h	18784516	9993324	51	9351613	93.2%	7172256
H8-48h	22175150	11562073	50.5	1080717	93.1%	7870190
H8-72h	19340153	9988254	50	9287351	92.6%	6542845

[a] Count of the total number of sequences processed.
[b] Number of sequences actually used for the rest of the analysis after quality filtering.
[c] Number of aligned pairs mapped to the annotated equine genome.
[d] Concordant pair alignment rate to the annotated equine genome.
[e] Counts of reads assigned to a gene feature in the annotation file.
