# Supplementary material for: Characterization of an Ex Vivo Equine Endometrial Tissue Culture Model Using Next-Generation RNA-Sequencing Technology
Source: Animals (Basel). 2021 Jul 3;11(7):1995. doi: 10.3390/ani11071995 (PMC8300099; doi:10.3390/ani11071995)

Figure S1

Pearson correlations between endometritis associated biomarkers genes focusing on (a) ESR1 and (b) PTGS2

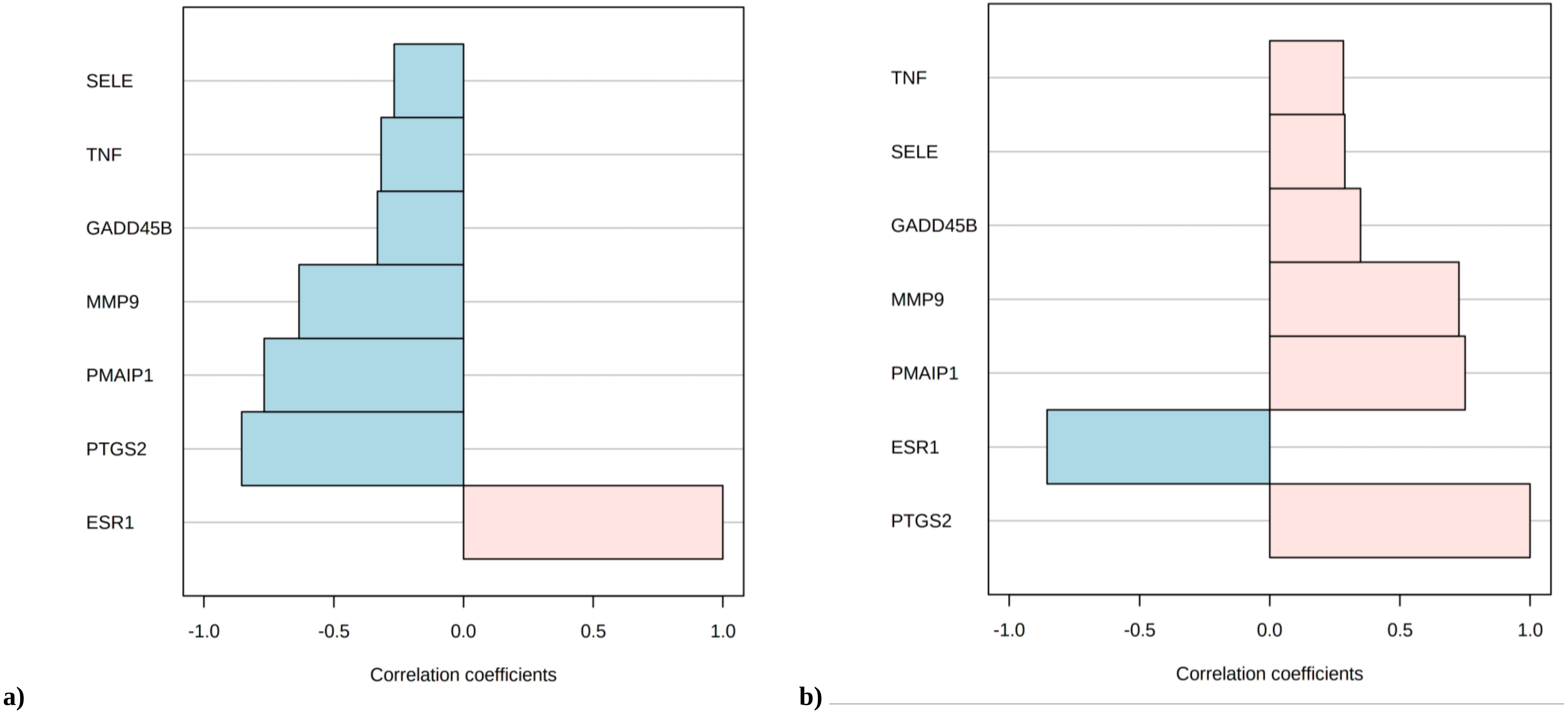

Supplement: Supplementary file 1 [file animals-11-01995-s001.zip › Supplementary File 5 - Figure S1 - Pearson correlations between endometritis associated biomarkers genes focusing on (a) ESR1 and (b) PTGS2.pdf]
