# Supplementary material for: Characterization of an Ex Vivo Equine Endometrial Tissue Culture Model Using Next-Generation RNA-Sequencing Technology
Source: Animals (Basel). 2021 Jul 3;11(7):1995. doi: 10.3390/ani11071995 (PMC8300099; doi:10.3390/ani11071995)
Supplement: Supplementary file 1 [file animals-11-01995-s001.zip › Supplementary File 6 - Figure S2 - DEGs in the Tumor Necrosis Factor TNF Signalling Pathway between 24 and 0 h.pdf]

Figure S2

DEGs in the Tumor necrosis factor (TNF) Signalling Pathway between 24 and 0 h (control)

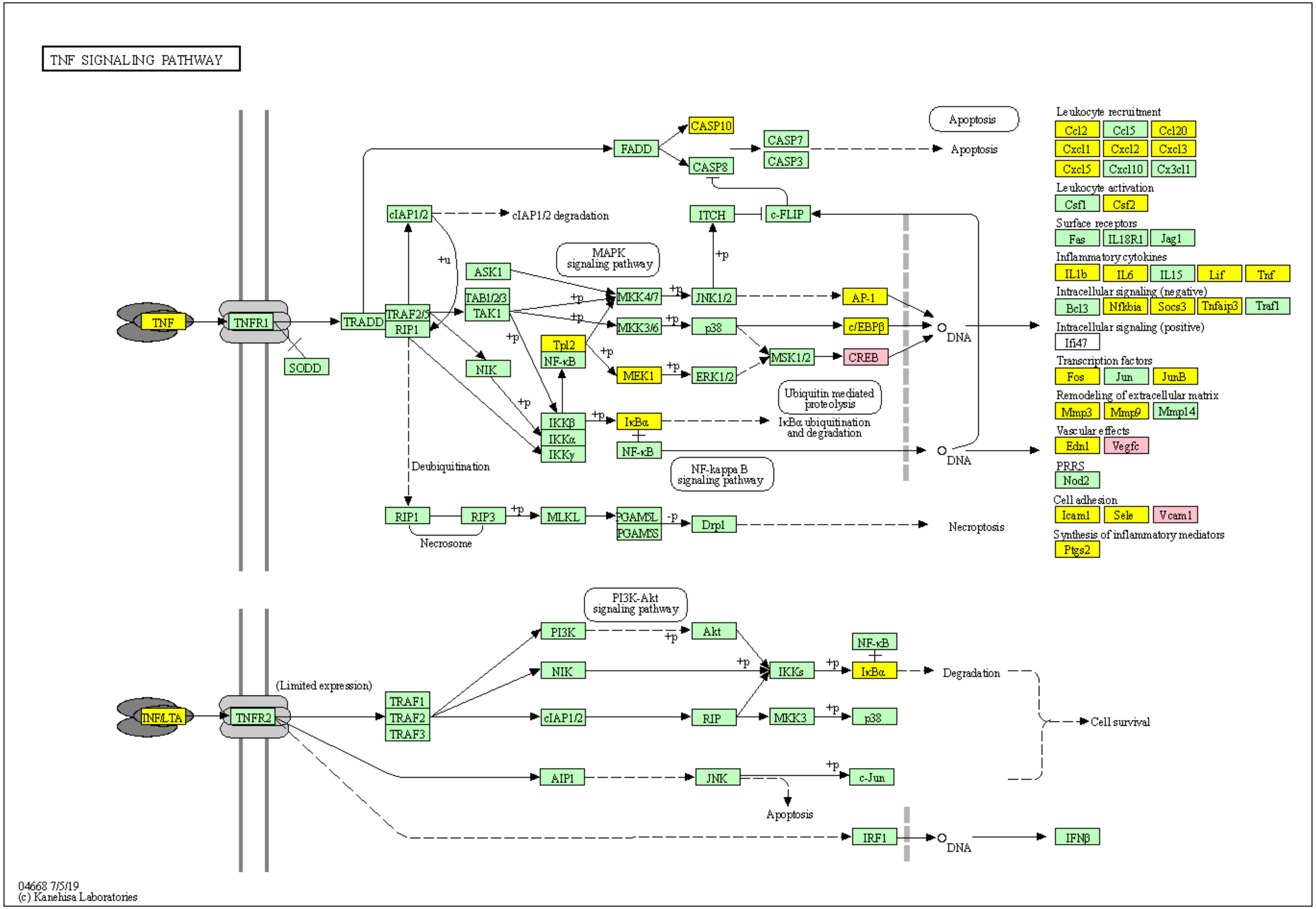

Green boxes are genes in the equine transcriptome, but not differentially expressed. White boxes are genes that are not found in the equine transcriptome. Yellow and pink boxes represent up- and downregulated DEGs respectively. Image created using the KEGG Online “Search and Color Pathway” tool.
