# Supplementary material for: Characterization of an Ex Vivo Equine Endometrial Tissue Culture Model Using Next-Generation RNA-Sequencing Technology
Source: Animals (Basel). 2021 Jul 3;11(7):1995. doi: 10.3390/ani11071995 (PMC8300099; doi:10.3390/ani11071995)
Supplement: Supplementary file 1 [file animals-11-01995-s001.zip › Supplementary File 7 - Figure S3 - DEGs in the Cytokine-Cytokine Receptor Interaction Pathway between 24 and 0 h.pdf]

Figure S3

DEGs in the Cytokine-Cytokine Receptor Interaction Pathway between 24 hours and 0 hours (control)

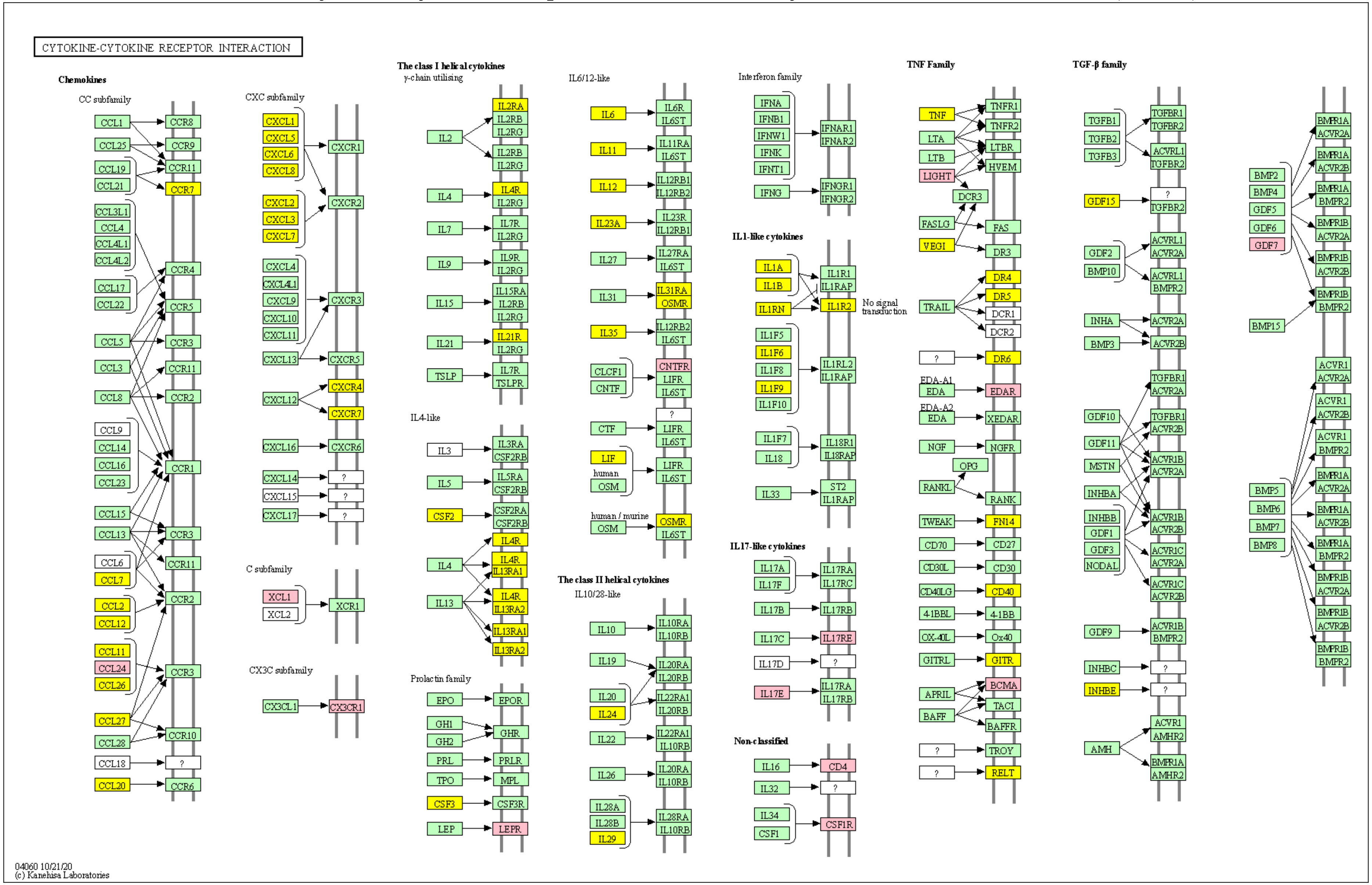

Green boxes are genes in the equine transcriptome, but not differentially expressed. White boxes are genes that are not found in the equine transcriptome. Yellow and pink boxes represent up- and downregulated DEGs respectively. Image created using the KEGG Online "Search and Color Pathway" tool.
